# Recent Advances in and Application of Fluorescent Microspheres for Multiple Nucleic Acid Detection

**DOI:** 10.3390/bios14060265

**Published:** 2024-05-22

**Authors:** Zhu Chen, Gaoming Luo, Jie Ren, Qixuan Wang, Xinping Zhao, Linyu Wei, Yue Wang, Yuan Liu, Yan Deng, Song Li

**Affiliations:** 1 MOE Key Lab of Rare Pediatric Diseases & Hengyang Medical School, University of South China, Hengyang 421001, China; luogaoming163@163.com (G.L.); rj759076926@163.com (J.R.); ww2287194534@163.com (Q.W.); zhaoxp921@163.com (X.Z.); wei_usc_3290bgd560@163.com (L.W.); gxuliuyuan@163.com (Y.L.); hndengyan@126.com (Y.D.); 2 Institute for Future Sciences, University of South China, Changsha 410008, China; 3 Hunan Province Key Laboratory of Tumor Cellular & Molecular Pathology, Hunan Engineering Research Center for Early Diagnosis and Treatment of Liver Cancer, Cancer Research Institute, Hengyang Medical School, University of South China, Hengyang 421001, China; 4 Institute of Cytology and Genetics, School of Basic Medical Sciences, Hengyang Medical School, University of South China, Hengyang 421001, China; 5 Hunan Key Laboratory of Biomedical Nanomaterials and Devices, Hunan University of Technology, Zhuzhou 412007, China; yuesir0029@163.com

**Keywords:** fluorescent microspheres, multiple nucleic acid detection, microsphere synthesis and modification, pathogens, tumors, genome

## Abstract

Traditional single nucleic acid assays can only detect one target while multiple nucleic acid assays can detect multiple targets simultaneously, providing comprehensive and accurate information. Fluorescent microspheres in multiplexed nucleic acid detection offer high sensitivity, specificity, multiplexing, flexibility, and scalability advantages, enabling precise, real-time results and supporting clinical diagnosis and research. However, multiplexed assays face challenges like complexity, costs, and sample handling issues. The review explores the recent advancements and applications of fluorescent microspheres in multiple nucleic acid detection. It discusses the versatility of fluorescent microspheres in various fields, such as disease diagnosis, drug screening, and personalized medicine. The review highlights the possibility of adjusting the performance of fluorescent microspheres by modifying concentrations and carrier forms, allowing for tailored applications. It emphasizes the potential of fluorescent microsphere technology in revolutionizing nucleic acid detection and advancing health, disease treatment, and medical research.

## 1. Introduction

The main purpose of nucleic acid detection technology is to detect and analyze nucleic acid sequences in samples to enable the detection and identification of biological processes such as gene mutations [1,2,3], viral infections [4,5,6], and targeted drug development [7,8,9]. Nucleic acid testing (NAT) for infectious diseases is used on a large scale. As bioassay technology continues to evolve, the need for objective testing continues to grow, and as the market demand increases, the use of a single nucleic acid test is no longer sufficient. For example, the 2019 coronavirus disease (*Neocoronavirus pneumonia*) has mutated [10,11,12,13], and there is an urgent need for simultaneous multitarget testing [14], multigene dosing, and multimarker testing [15]. Multiplex nucleic acid assays can be divided into multiplex polymerase chain reaction (PCR), multiplex nucleic acid hybridization (MNH), next-generation sequencing (NGS), multiplex nucleic acid amplification, mass spectrometry, CRISPR–Cas, microspheres, GeneChips, Raman scattering, etc. [16,17] Multiplex PCR enables simultaneous amplification of multiple target DNA fragments in a single reaction [18,19]. Multiplex in situ hybridization can determine nucleic acid localization in cells and tissues [20,21]. Furthermore, NGS can be used to determine DNA and RNA sequences in a high-throughput manner [22,23], while isothermal amplification of nucleic acids provides rapid amplification without temperature cycling [24,25]. However, multiplex PCR requires the design of multiple primers and optimization of reaction conditions, which increase the experimental complexity [26,27]. Multiple in situ hybridization, on the other hand, is demanding in terms of sample processing and signal detection and is characterized by background noise [28,29]. The disadvantages of NGS include high equipment and data analysis costs, as well as the requirement for sample preprocessing and library construction [30,31,32]. Nucleic acid isothermal amplification techniques are highly demanding in terms of primer design and amplification product analysis [33,34]. Mass spectrometry [35,36] and CRISPR–Cas technology [37,38] have advantages in nucleic acid detection, including high sensitivity, specificity, throughput, speed, and programmability. However, CRISPR has specific PAM/PFS sequence limitations for detecting genes and trans-shear activity, resulting in multiplexed assays that require multiple enzymes, RNA guide synthesis that is expensive and unstable in vitro, and limited signal differentiation for direct reads for single-base typing [39,40,41]. Moreover, mass spectrometry has the problems of high sample requirements, limitations for unknown nucleic acid sequences, low sensitivity, and high equipment and technical requirements [42,43]. While the application of nanotechnology offers the possibility of developing a new generation of nucleic acid detectors and biosensors [44,45,46], the preparation process is complex and expensive, and there are challenges related to the controllability and stability of these materials in practical applications [47,48]. Overall, however, these new technologies offer multiple advantages, including ease of use, efficiency and speed, high sensitivity, real-time monitoring, and visualization of results. Through continuous innovation and technological integration, scientists have been able to study the genome and diagnose diseases in greater depth, advancing the frontiers of biomedical and molecular biology.

Fluorescent microspheres, as an important tool in suspension array technology, are characterized by multiple fluorescent signals and tunable sizes and shapes, which provide strong support for the development of this technology [49,50,51]. Fluorescent microspheres are tiny particles with fluorescent properties whose surfaces are labeled with fluorescent substances or whose surfaces contain fluorescent substances. When stimulated by external energy, fluorescent microspheres can emit fluorescence [52]. A functional microsphere can have various shapes but is usually spherical. Fluorescent microspheres have a relatively stable morphological structure and luminescent behavior and are much less affected by external conditions such as solvents, heat, electricity, and magnetism than pure fluorescent compounds. This approach provides fluorescent microspheres with the important advantages of multichannel detection, high sensitivity and specificity, customizable labeling, low sample requirements, automation capabilities, and high-throughput detection in research and applications [53,54].

**Figure 1:**
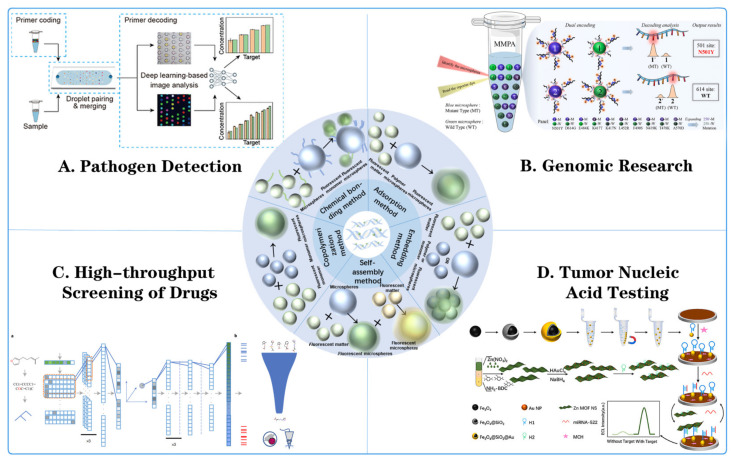
Fluorescent microsphere synthesis method and its application in multiple nucleic acid detection. (**A**) Pathogen detection: detecting pathogens through fluorescent microsphere encoding primers, droplet pairing and merging, primer decoding, and deep-learning-based image analysis [55]. (**B**) Genomic research: using microsphere nucleic acid probes (MNPAs) for target recognition, signal amplification, and detection [56]. (**C**) High-throughput screening of drugs: including drug library, fluorescence kinetics monitoring, and data analysis for drug screening. ((**a**) The molecules were first represented by SMILES, then parsed to a grammar tree, and then to a one-hot array, which was then passed to three layers of one-dimensional convolutional neural networks. The output slices were flattened and then passed to a dense layer, which output the mean vector and radius vector to encode a sphere in high-dimensional space. The coordinates of a randomly sampled point in this sphere were chosen as the latent vector, which was then passed through a five-layer dense network (dashed line indicates a dropout layer) for prediction of CTPs of 978 landmark genes. Finally, the 978 landmark genes were converted to 12,328 genes through linear transformation (see Methods for details of all layers). (**b**) Gene signatures of particular diseases of interest were employed to predict the efficacy of compounds using GSEA. Then virtual screening was performed against the selected chemical library. Efficacy was tested using cell lines or animal models directly, rather than through initial testing at the protein level.) [57]. (**D**) Tumor nucleic acid testing: detailed procedures for DNA extraction, amplification, and detection using fluorescent microspheres were demonstrated [58].

This paper focuses on fluorescent microspheres and reviews the latest research progress in their application in multiple nucleic acid detection, mainly including fluorescent microsphere synthesis and modification, fluorescent microsphere application in pathogen detection, genomics research, drug high-throughput screening, and tumor nucleic acid detection. This paper aims to provide a new opportunity for exploring the advantages of detecting multiple targets at the same time, as well as having high throughput, low cost, and other advantages, which can satisfy large-scale screenings, quantitative assays, and other assays. It is expected that this approach can provide a new insight and method for solving the abovementioned problems (Figure 1).

## 2. Fluorescent Microsphere Synthesis and Modification

### 2.1. Methods for the Preparation of Fluorescent Microspheres

**Figure 2:**
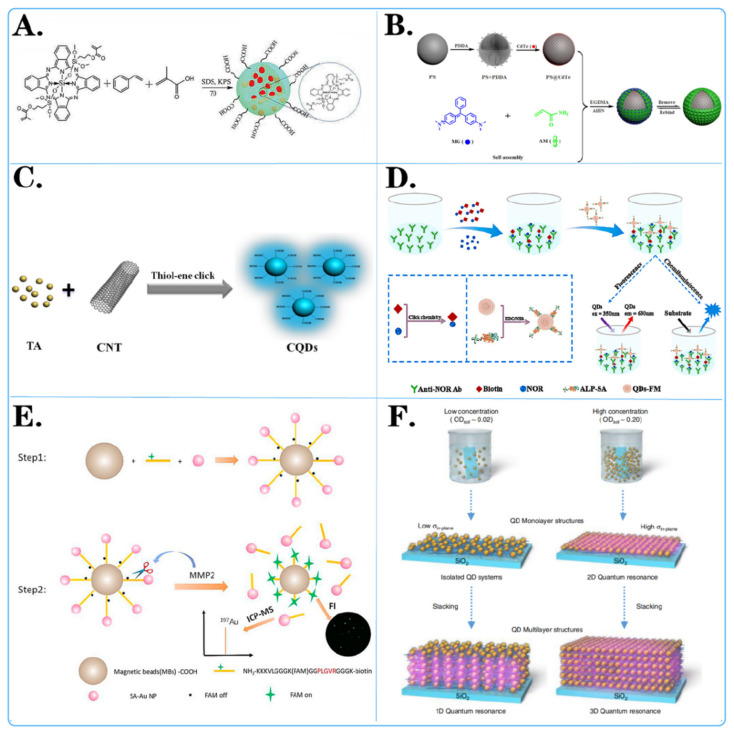
Surface modification method of fluorescent microspheres. (**A**,**C**) Chemical modification of fluorescent microspheres: showcased is the chemical structure of molecules attached to the surface of microspheres. (**D**,**E**) Biological modification of fluorescent microspheres: displayed are various biochemical reactions and interactions on the surface of microspheres. (**B**,**F**) Physical modification of fluorescent microspheres: displayed is the layered adhesion of molecules on the surface of microspheres through methods such as charge modification and magnetic modification.

Current literature reports a variety of methods for preparing fluorescent microspheres that can be selected on demand (Table 1). The more common methods include adsorption, embedding, self-assembly, chemical bonding, and copolymerization (Figure 2).

#### 2.1.1. Adsorption Method

The adsorption method involves mixing an organic solution containing a fluorescent substance with an aqueous dispersion system of microspheres and then loading the fluorescent substance onto the surface of the microspheres through physical adsorption to produce fluorescent microspheres [59].

Jiangxin Liu and colleagues [60] proposed a strategy to prepare fluorescence-encoded microspheres using two hydrophobic polymers, polystyrene (PPE) and Nile red (NR), as monodisperse amino-modified porous matrix polymer spheres. The fluorescent dyes were loaded into the APGMA-PPE-NR microspheres by impregnation and adsorption using poly(glycerol polymethacrylate-dimethacrylate) (APGMA) as the carrier to form the APGMA-PPE-NR microspheres. A total of 64 different coded APGMA-PPE-NR microspheres were easily obtained by simply adjusting the concentration and combination of the two agents. Jingxin Zhou and colleagues [61] used oxidative degradation and ionic crosslinking techniques to prepare chitosan nanoparticles for the study of the drug-carrying properties of quercetin. Uniform chitosan-quercetin (CS-QT) drug-carrying nanoparticles were made by adding appropriate amounts of quercetin-anhydrous ethanol solution and fluorescein isothiocyanate (FTIC) to chitosan nanoparticle (CSNP)-acetic acid solution. The results showed that the fluorescently labeled nanosystems exhibited excellent antimicrobial properties, which provided a strategy for observing drug release and functionality. Vasanthakumar and others [62] prepared a novel composite consisting of multiwalled-carbon-nanotube (MWCNT)-loaded V_2_O_5_ quantum dots modified with Bi_2_O_3_ hybrids by a simple wet impregnation method and investigated the photocatalytic performance of the prepared samples for the photodegradation of ciprofloxacin (CIP). Photocatalytic tests showed that the MWCNT@V_2_O_5_/Bi_2_O_3_-5 hybrid composites exhibited enhanced photocatalytic activity for CIP degradation compared to that of pure photocatalysts and other photocatalysts. This work provides a new approach for designing efficient photocatalysts for pollutant degradation.

#### 2.1.2. Embedding Method

The embedding method disperses the fluorescent material uniformly in the synthesis system of carrier microspheres and subsequently aggregates the fluorescent material into spheres through the polymerization of monomers or microencapsulation of the carrier matrix material, so that the fluorescent material is naturally embedded in the interior of the microspheres [63].

Wan-Sheng Tang and colleagues [64] prepared quantum dot-encoded maleic anhydride-grafted PLA (PLA-MA) fluorescent microspheres by embedding CdSe/ZnS quantum dots in PLA-MA using the Shirasu porous glass (SPG) membrane emulsification technique. The microspheres were characterized by high fluorescence intensity, good stability, and good dispersion. Fluorescence immunoassay of the test strips revealed that the PLA-MA microspheres have high bioactivity and good stability, which is favorable for immunoassays. Qiaoli Jin and others [65] first synthesized 13 nm CuInZnS/ZnS QDs with DDT ligands, and then CuInZnS/ZnS microbeads (QBs) containing thousands of QDs were successfully prepared by a two-step method of emulsion-solvent evaporation and surfactant substitution. By emulsion-solvent evaporation, CuInZnS/ZnS QDs were formed into microbeads in a microemulsion with dodecyltrimethylammonium bromide (DTAB), and Förster resonance energy transfer (FRET) was effectively overcome. Then, CO-520 was introduced to replace DTAB to improve its stability and water solubility. Finally, highly fluorescent and stable CuInZnS/ZnS QBs were successfully constructed by coating the microbeads with SiO_2_ shells and carboxylating them. Yaofeng Zhou and colleagues [66], on the other hand, dissolved octadecylamine-coated CdSe/ZnS QDs in aqueous solutions of cyclohexane and SDS and emulsified them with an ultrasonic generator before evaporation and centrifugation, followed by the addition of NaHCO_3_ solution overnight. Finally, purified carboxylated quantum dot nanobeads (QBs) were obtained by centrifugation.

#### 2.1.3. Self-Assembly Method

The self-assembly method utilizes inorganic or organic particles as a substrate or core, which are assembled by the interaction force between the particles. In this process, fluorescent materials are embedded inside the microspheres to form microsphere structures with fluorescent properties.

Tian Qiu and colleagues [67] synthesized multilayer fluorescent microspheres using a layer-by-layer self-assembly method by using polystyrene-divinylbenzene (SPSDVB) microspheres with negative sulfonic acid groups on the surface as matrix microspheres and poly(p-styrene) as a fluorescent material; these microspheres had good morphology and strong fluorescence. Chongwen Wang’s research team [68] proposed a novel approach to prepare core-shell magnetic quantum dots (Fe_3_0_4_@DQDs) by using polyethyleneimine (PEI)-mediated layer-by-layer self-assembly. These QDs have a double-quantum-dot shell-layer structure with monodispersity, high magnetic responsiveness, good stability, and excellent fluorescence properties. Hanyu Wang and colleagues [69] prepared carbon dots by a one-pot low-temperature hydrothermal method and cellulose-based fluorescent microspheres by an electrostatic self-assembly green method. The adsorbents were prepared via a simple process with good reproducibility, high adsorption capacity, and versatility, without the addition of toxic small-molecule cross-linking agents or artificial polymers.

#### 2.1.4. Chemical Bonding Method

The chemical bonding method utilizes the formation of chemical bonds between the active groups of the fluorescent molecules and the active groups of the microspheres to achieve immobilization of the fluorescent molecules and stable attachment to the surface of the microspheres.

Leire San José and colleagues [70] successfully synthesized fluorescent hybrid ZnO quantum dots with sizes between 4 and 5 nm using the sol-gel method and different hydroxyl polymers as templates and ligands. Low-dispersion polyhydroxylated polymers and block copolymers were obtained by reversible addition fracture chain transfer (RAFT) polymerization to determine the influence of molecular weight, hydrophobic/hydrophilic equilibrium, and polymer structure on the colloidal and photophysical properties of the nanohybrids of ZnO quantum dots. Fluorescence enhancement occurs when ZnO QDs are synthesized in the presence of hydroxylated polymers, especially when block copolymers are used. Lijuan Sun and colleagues [71] proposed a method to prepare monodisperse fluorescent microspheres with active sites by introducing another poly(p-styrene) conjugated polymer with carboxyl groups. Two poly(p-styrene) (PEE) polymers (CP1 and CP2) were synthesized by Sonogashira, a coupling followed by hydrolysis of the ester group. The carboxyl groups are activated, and the activated CPs are subsequently immobilized in the spheres through covalent bonding to produce fluorescent microspheres with reactive sites. Panpan Pan and others [72] synthesized bifunctional core-shell magnetic fluorescent microspheres by a facile interfacial Pechini-type sol-gel method using citric acid and polyethylene glycol as chelating and cross-linking agents, respectively. The obtained Fe_3_O_4_@YVO4:Eu^3+^ microspheres had a typical core-shell structure, high magnetization intensity, and strong fluorescence emission.

#### 2.1.5. Copolymerization Method

The copolymerization method covalently bonds fluorescent molecules into fluorescent microspheres by polymerizing fluorescent molecules with polymerizable functional groups with organic monomers with polymerizable functional groups. In this method, fluorescent molecules and organic monomers are tightly bound together via covalent bonds, resulting in a uniform distribution of fluorescent molecules in the microspheres [73].

Wang and colleagues [74] used a stepwise self-stabilizing precipitation polymerization method to construct a novel material called WLEP by combining two AIE atoms with fluorescent complements. This material has AIE properties and undergoes FRET between core-shell fluorescent polymer particles (CS-FPPs), which can change the microenvironment of the aggregated state by regulating the expansion and contraction of the polymer, thus realizing tunable white light fluorescence in response to CS-FPPs. Xiaoqin Liang’s research team [75], on the other hand, successfully prepared a material called polymerization-induced emission (AIE) polymer nanoparticles (PNPs) by fine emulsion polymerization of tetraphenylene (TPE), a molecule with AIE properties, encapsulated in a polymer matrix. Tugrul Cem Bicak and others [76] reported a simple and cost-effective method for the synthesis of fluorescent microspheres without the need for any fluorescent monomers or modification steps to dope the fluorescent portion into polymer particles. Using rhodamine B and benzophenone as a bimolecular initiation system for type II photoinitiated precipitation polymerization, fluorescent microspheres were prepared in a single step at room temperature without the need for any type of stabilizer or surfactant.

In summary, the adsorption method is relatively simple and straightforward to operate, although it has poor stability. The embedding method can maintain the stability of the pigment, but the operation is complex. The self-assembly method has high efficiency and controllability but requires specific conditions. The chemical bonding method has high stability, but the operation is complex and may affect the properties of pigments. The copolymerisation method can regulate the properties of pigments but requires suitable copolymers.

### 2.2. Surface Modification Method for Fluorescent Microspheres

Surface modification methods for fluorescent microspheres can improve their stability, optical properties, and biocompatibility by changing their surface properties. Common modification methods include chemical modification, biological modification, and physical modification (Figure 2).

#### 2.2.1. Chemical Modification Method

Chemical modification methods for fluorescent microspheres include covalent modification and polymer coating. Covalent modification introduces different functional groups, such as carboxyl, amino, and phosphate groups, to the surface of fluorescent microspheres through chemical reactions to covalently bind with other molecules (e.g., antibodies, drugs, or dyes) to immobilize them on the surface of the microspheres for specific functions. Polymer coatings, on the other hand, include coatings, such as polyethyleneimine (PEI) or polyvinyl alcohol (PVA), to improve the stability and biocompatibility of the microspheres. These methods impart diverse properties and applications to fluorescent microspheres, such as altering the hydrophilicity and charge of the surface. Commonly used chemical modification methods include carboxylation reactions, amination reactions, and sulfation reactions.

For example, Dan-Dan Liu and colleagues [77] employed novel vinylsilicon phthalocyanine, styrene (St), and methacrylic acid as monomers, with potassium persulfate as the initiator, to synthesize carboxylated silicon phthalocyanine photosensitive microspheres in situ (Figure 2A). The experimental results indicated that silicon phthalocyanine molecules with flexible unsaturated side chains exhibited excellent compatibility with styrene. The presence of carboxyl groups resulted in highly monodisperse and stable photosensitive microspheres. Ziyang He and colleagues [78] developed a simple, cost-effective, and direct strategy to prepare fluorescent carbon quantum dots (CQDs) through a one-pot thiol-ene click reaction between multiwalled carbon nanotubes (CNTs) and thioacetic acid (TA) (Figure 2C). Various characterization data confirmed the successful synthesis of CQD. The results demonstrated that CQD effectively combined with TA through surface thiol-ene click chemistry. Pablo G. Argudo et al. [79] synthesized fluorinated quantum dots from ZnCdS/ZnS QDs sealed with trioctylphosphine oxide (TOPO) and the fluorinated ligand HS-C_11_-(EG)_4_-O-C(CF_3_)_3_. It was experimentally demonstrated that fluorination of nanoparticles can be used as a simple and general strategy to promote the interaction of nanoparticles with cell membranes and to imply their effective cellular uptake. This work opens new possibilities for designing NP-based biomedical tools for diagnosis and therapy, as well as improving cell uptake and, therefore, potentially improving performance.

#### 2.2.2. Biological Modification Method

Biological modifications of fluorescent microspheres include the biotin–streptavidin system, which utilizes biotin–streptavidin interactions to attach biologically active molecules (e.g., proteins or nucleic acids) to the surface of the microspheres, as well as antibody conjugation, which allows specific cells or biomolecules to be targeted through the binding of specific antibodies to the microspheres. These methods make fluorescent microspheres biocompatible and biospecific.

For example, Xiao Liu and colleagues [80] developed a dual-read immunoassay for rapid and sensitive detection of norfloxacin based on QDS-FM@Alp-SA and click chemistry (Figure 2D). In this system, a biotin conjugate (biotin) was synthesized using click chemistry for signal conversion. Alkaline-phosphatase-labeled streptavidin (ALP-SA) was attached to quantum dot fluorescent microspheres (QDS-FMs) via active ester chemistry, creating QDS-FM@ALP-SA for signal amplification. Xiaoting Li and colleagues [81] designed a dual-functional magnetic microsphere probe for ICP–MS quantification and fluorescence imaging of MMP2 during cellular secretion (Figure 2E). In this designed probe, NH_2_-peptide (FAM)-biotin served as a bridge between carboxylated magnetic beads (MBs-COOH) and streptavidin-functionalized gold nanoparticles (Au NP-SAs). Initially, the fluorescence of FAM was quenched by the Au NPs. Since the NH_2_ peptide (FAM)-biotin contains an MMP2-specific recognition sequence, this peptide was specifically cleaved in the presence of MMP2, which released Au NPs for MMP2 quantification by ICP–MS and restored the fluorescence of FAM for MMP2 fluorescence imaging. Huanan Wang et al. [82] conjugated monoclonal antibodies specific to the ALV main capsid protein encoded by the gag gene, labeled fluorescent microspheres and coated ALV antibodies on nitrate cellulose membranes to prepare test lines for sample detection. A simple, rapid, sensitive, and specific immunochromatographic test strip based on a specific monoclonal antibody against the capsid protein p27 was successfully developed.

#### 2.2.3. Physical Modification Method

Physical modification methods for fluorescent microspheres include charge modification and magnetic modification. Charge modification achieves electrostatic attraction or repulsion effects by changing the charge characteristics of the microsphere surface for particle dispersion or separation. Magnetic modification, on the other hand, involves binding magnetic particles to microspheres to control their position and movement under an external magnetic field, extending their range of applications, with particular potential for the separation and manipulation of magnetically guided materials. These physical modification methods add additional functionality and manipulation capabilities to fluorescent microspheres for a wide range of scientific applications.

For instance, Ji Yang and colleagues [83] achieved amplification of fluorescent signals by electrostatic self-assembly of CdTe quantum dots on the surface of polystyrene (PS) microspheres (Figure 2B). They prepared the MIP@PS@CdTe fluorescent probe after grafting molecularly imprinted membranes and applied it to detect MG (malachite green) based on fluorescence quenching. The detection limit for MG was significantly lower than previously reported limits, highlighting the accuracy and reliability of the MIP@PS@CdTe probe. TaeGi Lee and colleagues [84] constructed a CdTe quantum dot superlattice through the layer-by-layer assembly of positively charged polyelectrolytes and negatively charged CdTe quantum dots (Figure 2F). They controlled the dimensions of quantum resonances by independently changing the distances between quantum dots in the out-of-plane and in-plane directions. The formation of microbands was experimentally verified by measuring the excitation energy dependence of the photoluminescence spectra and the detection energy dependence of the photoluminescence excitation spectra.

In the preparation of fluorescent microspheres, chemical modification methods typically necessitate the acquisition of costly chemical reagents and materials, intricate operations, and high technical standards. However, they can achieve precise surface control and regulate the properties and functions of the microspheres with high sensitivity and versatility. Biological modification methods may involve expensive biological reagents or enzymes and necessitate the utilization of sophisticated biological experimental techniques and are more challenging to prepare. Nevertheless, they can achieve bioactive modification and enhance the biocompatibility and bioactivity of microspheres. Physical modification methods are relatively simple, less expensive, and less difficult to prepare. However, the functional modification of microspheres is limited, and the functional change is mainly achieved by modulating the surface morphology. Therefore, when choosing a suitable modification method, various factors need to be considered according to the specific research purpose and requirement.

## 3. Applications of Fluorescent Microspheres in Multiplex Nucleic Acid Detection

Fluorescent microspheres, as an emerging technology in multiplex nucleic acid detection, hold great promise for clinical diagnostics. These methods have wide-ranging applications in pathogen detection, genomics, drug screening research, and tumor nucleic acid detection, among other areas.

### 3.1. Application of Fluorescent Microspheres in Pathogen Detection

**Figure 3:**
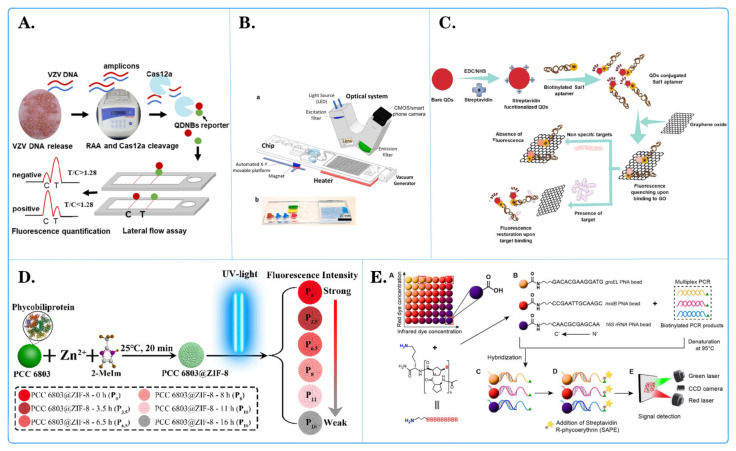
Fluorescent microspheres applied for pathogen detection. (**A**) Schematic diagram of quantum dot nanobeads (QDNB), labeled side flow analysis for the detection of varicella zoster virus (VZV) [86]. (**B**) Schematic diagram of a fully integrated sample in answer out platform for virtual detection using digital reverse transcription recombine polymer amplification (dRT RPA). ((**a**) Schematic illustration of the total system consisting of a magnetic platform, heater unit, vacuum generator, detection part with camera, and light-emitting diode (LED). The disposable chip has three operational zones to prepare and mix the reagents and detect the pathogen agent. (**b**) Dye-loaded chambers for visualization of chips. Brown, blue, and red show lysis, washing and elution chambers, respectively, for sample preparation. Green and yellow chambers show RPA mixture and mineral oil chambers, respectively.) [89]. (**C**) Schematic diagram of a simple and multifunctional aptamer sensor based on fluorescence resonance energy transfer (FRET) [87]. (**D**) Schematic diagram of light-regulated natural fluorescence of the PCC 6803@ZIF-8 composite as an encoded microsphere for the detection of multiple biomarkers [88]. (**E**) Schematic diagram of Bacillus cereus detection microsphere array technology based on peptide nucleic acid. ((**A**) a carboxyl group on each fluorescently barcoded paramagnetic bead allows covalent conjugation with the amino group on the acpcPNA molecule. (**B**) Three bead sets coupled with three specific acpcPNAs to groEL, motB and 16S rRNA genes were used to detect biotinylated PCR products from a multiplex PCR method. (**C**) Biotinylated single-stranded DNA after denatured at 95 °C was hybridized to acpcPNA-based beads. (**D**) R-phycoerythrin-labeled streptavidin (SAPE) molecules were bound to the biotin tag on the DNA-PNA bead complex. (**E**) A green laser was used to detect fluorescent signal from SAPE and a red laser was used to identify the region of bead set.) [90].

Fluorescent microspheres can be used to detect nucleic acid sequences of a variety of pathogens, including bacteria, viruses, and fungi (Table 2). By combining fluorescent microspheres with specific pathogen nucleic acid sequences, the presence of the pathogen in a sample can be rapidly and accurately detected, and disease diagnosis and monitoring can be performed (Figure 3).

**Table 2:** Application of fluorescent microspheres for pathogen detection.

Microsphere Type	Detection System	Detection Object	Advantage	Document Source
Magnetic microspheres with anti-TAG oligonucleotide coupling	Luminex xTAG	PKoV, PAstV, PEDV, PSaV, PSV, PTV, PDCoV, TGEV, BVDV, PoRV, and PToV virus DNA	More sensitive Better specificity	[85]
Quantum dot nanobeads (QDNB)	Side-flow analysis (CQ-LFA)	VZV virus DNA	Rapid decision making Cheaper costs Clinical applications	[86]
Quantum dots (QD)	A simple and versatile aptamer sensor based on fluorescence resonance energy transfer (FRET)	ssDNA of *Salmonella paratyphi* A	Simple design High specificity High sensitivity	[87]
Fluorescent PCC 6803@ZIF-8	A bead-based assay platform	DNA insertion sequence of *Mycobacterium tuberculosis*	Simplifies protein fixation procedures Reduces non-specific adsorption High sensitivity Eliminates the chemically synthesized fluorescent materials	[88]
RNA-bound magnetic beads	Digital reverse transcription recombinase polymerase amplification (DRT-RPA)	RNA of SARS-CoV-2	Fully automated virus diagnostics Minimized controller use No interference with isolation and amplification steps Cheap, simple, and accurate digital RT-RPA implementation	[89]
#MC10012, #MC10015, #MC10021, Luminex	Microbead array method	DNA of *Bacillus cereus*	High methodological robustness Minimal food interference Suitable for a wide range of food samples	[90]
Polystyrene microsphere	Fluorescence encoding method	DNA sequences of COVID-19 (with related mutations)	Compatible with the latest fluorescence microscopes Easy coding and decoding Suitable for multiple high-throughput analyses	[91]

Ying Shi and team [85] utilized the Luminex xTAG multiplex detection method based on fluorescent microspheres to detect 11 viral diarrheal pathogens, namely, PKoV, PAstV, PEDV, PSaV, PSV, PTV, PDCoV, TGEV, BVDV, PoRV, and PToV. The results revealed that PKoV, PAstV, and PEDV were the most common viruses in diarrheal pigs, with detection rates of 38.65% (291/753), 20.32% (153/753), and 15.54% (117/753), respectively. This study contributes to a better understanding of viral diarrheal pathogens in piglets, aiding in the prevention and control of viral piglet diarrhea outbreaks. Xiaoqin Zhong and colleagues [86] developed a quantum dot nanobead (QDNB)-labeled lateral flow assay for the detection of varicella-zoster virus (VZV), which is capable of identifying more than five VZV genomic DNA copies per reaction (Figure 3A). The entire process, from sample preparation to obtaining results, took less than one hour. Importantly, this method outperforms clinical diagnosis when vesicles appear in specific areas (e.g., the genital region). Compared to traditional detection methods, only a small amount of vesicle fluid is needed for accurate testing, making this method a potential candidate for clinical application in specific and rapid VZV detection. Renuka and collaborators [87] developed a simple and multifunctional aptamer sensor based on fluorescence resonance energy transfer (FRET) for the rapid detection of Salmonella typhi (Figure 3C). Compared to traditional immunological assays such as ELISA, FRET-based aptamer assays excel in terms of specificity, speed, reliability, and simplicity. Therefore, the proposed method can be used for target pathogen detection through FRET-based assays. They used classical molecular dynamics simulations to explore further interactions between aptamers and the pathogenic protein DNA gyrase. Yuqiang Xiang and his team [88] assembled fluorescent PCC 6803@ZIF-8 composite materials as a bead-based assay platform for *Mycobacterium tuberculosis* detection via a self-assembling zeolitic imidazolate framework (ZIF-8) on the surface of inactivated PCC 6803 cells (Figure 3D). Due to the presence of pigment proteins in PCC 6803, the composite fluorescence gradually changed to white with prolonged exposure to ultraviolet light. Consequently, composite materials with different fluorescence intensities were obtained as encoding microspheres for multiplex detection. ZIF-8 provided stable, rigid outer shells and a large specific surface area to the composite material, preventing damage during use and storage, simplifying protein fixation procedures, reducing nonspecific adsorption, and enhancing detection sensitivity. The encoded composite materials were successfully used to detect multiple DNA insertion sequences in *M. tuberculosis*. The proposed strategy provides an innovative color encoding method for high-throughput multiplex biotests without the use of chemically synthesized fluorescent materials. Islam Seder and colleagues [89] simplified isothermal amplification technology and employed a one-step digital reverse transcription–polymerase chain reaction (dRT-RPA) technique for virus RNA detection, enabling rapid diagnosis and absolute quantification (Figure 3B). The proposed chip can automate the purification, digitization, and detection of SARS-CoV-2 RNA through magnetic bead–RNA binding and digital amplification. The chip integrates magnetic valve and vacuum systems and can complete absolute quantitative detection of viral RNA in approximately 37 min, with a sensitivity as low as 10 RNA copies/μL. Consequently, this chip holds potential as an automated and accurate tool for infectious disease diagnosis. Prae Noppakuadrittidej and colleagues [90] researched a reliable detection method for *Bacillus cereus* using highly specific pyrrole-imidazole (acpcPNA) probes in combination with three sets of fluorescent barcoded beads (Figure 3E). This acpcPNA-based bead array exhibited exceptional selectivity, generating a signal only in the presence of *B. cereus*, without interference from other species. The method displayed high sensitivity for DNA detection of the groEL, motB, and 16S rRNA genes, surpassing their DNA counterparts, confirming the binding strength of acpcPNA. The reliability of the method was further validated through testing in food samples, indicating the effectiveness of the acpcPNA-based bead array as an alternative nucleic acid method for foodborne pathogen detection. Chao Guo and his colleagues [91] proposed a fluorescence encoding method with a light switch in a monochromatic channel. It utilizes photochromic naphthopyran to induce fluorescence in polystyrene microspheres through resonance energy transfer. The combination of initial fluorescence intensity (F0) and fluorescence after UV activation (F/F0) generates hundreds of two-dimensional barcodes. With different photoswitching chemical kinetics, the encoding capacity is further expanded. Light-switch-based fluorescence barcodes are used for the selective detection of COVID-19 DNA sequences (with relevant mutations) as a proof of concept for practical applications. This method is compatible with state-of-the-art fluorescence microscopy, and the encoding and decoding processes are simple, making it highly attractive for multianalysis and high-throughput analysis.

A comparison of the above fluorescent microspheres in different pathogens reveals the potential and application advantages of fluorescent microspheres in detecting different pathogens. Studies have demonstrated that the fluorescent microsphere method offers multiple detection capabilities, high sensitivity, rapidity, and specificity. It can simultaneously detect a wide range of pathogens, has high sensitivity for very small numbers of pathogens, and can complete the detection in a short period of time with high specificity. Nevertheless, the fluorescent microsphere method still faces limitations, including complex operation and poor stability in its application. Therefore, future research should focus on optimizing the fluorescent microsphere assay, improving its cost-effectiveness, simplifying the operation process, enhancing stability, and expanding its application in clinical diagnosis and disease control. These efforts will help to promote the further development and application of fluorescent microsphere technology in the field of pathogen detection.

### 3.2. Application of Fluorescent Microspheres in Genomic Research

**Figure 4:**
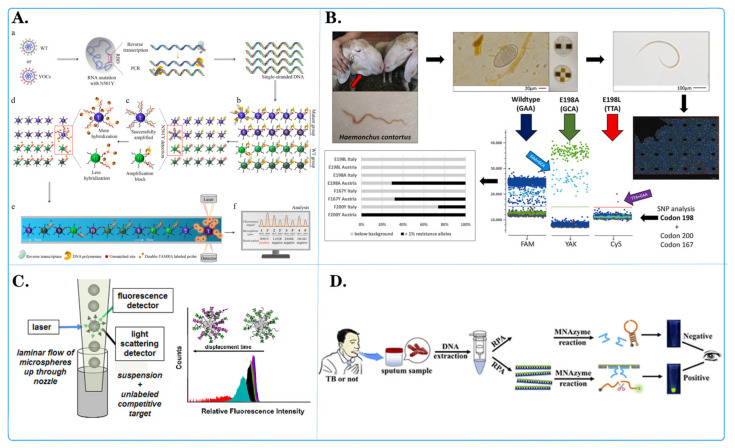
Application of fluorescent microspheres in genomics research. (**A**) Schematic diagram of an encoder-based multi-channel low light level phase amplification (MMP) sensing platform. ((**a**) Following asymmetric PCR amplification, a large amount of single-stranded DNA was generated. (**b**) Used in the MMPA assay with 20 primer-coated distinct microspheres in one reaction tube. (**c**) Following MMPA, mutant-coated microspheres produced more amplification products than wild-type (WT)-coated microspheres. (**d**) The amplified products were hybridized with dual-labeled fluorescent reporter probes. (**e**) Detected using a Luminex 200. (**f**) The fluorescence levels on the surface of wild-type- and mutant-coated microspheres were compared to determine the N501Y mutation). [56]. (**B**) Schematic diagram of development of a three-color digital PCR for early and quantitative detection of benzimidazole-resistance-associated single nucleoside polymorphisms in Haemonchus contortus [92]. (**C**) Schematic diagram of quantitative analysis of in situ locked nuclear acid and DNA competitive displacement events on microspheres [94]. (**D**) Schematic diagram of a multicomponent nuclear acid enzyme-clearable quantum dot nanobeacon for highly sensitive diagnosis of tuberculosis with the naked eye [93].

Fluorescent microspheres have a wide range of applications in genomics research and can be used for genotype analysis, gene expression analysis, genome rearrangement analysis, and genome copy number variation analysis (Figure 4). Their use can provide more accurate and high-throughput data, contributing to a deeper understanding of the structure and function of the genome.

Zecheng Zhong and colleagues [56] introduced a programmable multilane microsphere-based multiplex microsphere phase amplification (MMPA) sensing platform that combines microsphere technology with a dual fluorescence decoding strategy to detect SARS-CoV-2 RNA while identifying 10 key SARS-CoV-related viruses in the receptor-binding domain (RBD) (Figure 4A). Through repeated experiments, the straightforward design of the MMPA can be quickly modified to target any newly emerging infectious disease, and the MMPA has the potential to detect various types of pathogens. Barbara Hinney and her team [92] developed and validated a detection method using three-color droplet digital PCR (dPCR) and chip technology to detect the major SNPs (F167Y, E198A, F200Y) associated with benzimidazole (BZ) resistance in *Hemonchus contortus* (Figure 4D). The detection limit for this method was 1% allele frequency. The experimental results demonstrated that the developed dPCR detection method enables highly sensitive and accurate detection of major BZ-associated SNPs related to *H. contortus* resistance. Ou Hu and colleagues [93] invented a new platform for the rapid, simple, and sensitive colorimetric detection of tuberculosis (Figure 4C). This platform is based on quantum dots and a multicomponent nucleic acid enzyme (Mnazyme) and employs recombinase polymerase amplification, chemical modification, and the Mnazyme reaction to detect target DNA (the IS1081 gene segment) through QD-NB fluorescent probes and hybridization. Upon the addition of RNA as a separable substrate, QD-NB are cleaved into two DNA segments, resulting in the release of green fluorescence. This Mnazyme colorimetric method based on QD-NB offers increased sensitivity for tuberculosis detection and can be performed with the naked eye under a portable and inexpensive UV flashlight. The method exhibited excellent specificity and repeatability and was validated in clinical tuberculosis patients and healthy individuals, confirming its practical application in tuberculosis diagnosis. Ngozi A Eze and Valeria T Milam [94] employed high-throughput flow cytometry to immobilize probe strands on microspheres, measuring competitive displacement of the primary target through hairpin-mediated soluble fluorescent labeling (Figure 4B). They used LNA-DNA hybrid sequences and pure DNA sequences as fixed chains, along with labeled primary targets and unlabeled secondary or competing targets. They explored differences between chemically substituted sequences and unsubstituted sequences, as well as the effect of mismatched primary targets and holding segment positions on displacement profiles. The results revealed relatively mild displacement of the primary target and different responses to various double-stranded probe systems by the unlabeled secondary target. This research provides a new method for the quantitative analysis of locked nucleic acid (LNA) hybridization activity and reveals the characteristics of LNAs in nucleic acid competitive displacement. Stefania Ottaviani and her team [95] developed a novel diagnostic gene typing kit based on Luminex technology for 14 defective variants of the SERPINA1 gene, which showed 100% correlation with the results of the diagnostic gene typing kit when analyzing suspected α-1-antitrypsin deficiency (AATD) samples. The A1AT gene typing detection method is highly reliable and robust, significantly decreasing the time needed for diagnosis. A1ATGG analysis is a rapid and effective way to diagnose most defective variants. Muhammad G Kibriya [96] utilized a Luminex-based method to measure changes in relative telomere length (RTL) to study its association with different histological and molecular characteristics of colorectal cancer (CRC). The results showed that the RTL in CRC tissue was shorter than that in paired normal tissue (RTL 0.722 ± SD 0.277 vs. 0.809 ± SD 0.242, *p* = 0.00012). RTL shortening in CRC was associated with (a) upregulation of DNA replication genes and cyclin-dependent kinase genes (tumor suppressors) and (b) downregulation of the “cystathionine beta-synthase executor”, which is related to reduced apoptosis. Martin Ashby and others [97] used fluorescent-bead-based Luminex xMAP technology to simultaneously detect bluetongue virus (BTV) serotypes BTV-1 to BTV-24. After testing the reference strains, the xMAP assay was used to detect all 24 BTV serotypes. Overall, this assay offers a useful new diagnostic tool, especially for analyzing large sample sets. The use of BTV xMAP detection allows for rapid assessment of BTV epidemiology and may provide information for decision-making related to control and prevention measures. Yong Luo et al. [98] developed an ultrasound-enhanced catalytic hairpin assembly (UECHA) biosensing platform for effective biomarker enrichment and fluorescence enhancement in the early screening of Alzheimer’s disease. By introducing a portable acoustic driving platform and functionalized microspheres, the platform concentrates the functionalized microspheres in the center of the microcavity, accompanied by enhanced fluorescence signals with specific release. In addition, programmable frequency modulation can also modulate the acoustic potential well, effectively promoting nonequilibrium chemical reactions such as CHA (25 min). Compared with traditional catalytic hairpin assembly (CHA) components, UECHA allows for direct and quantitative measurement of AD miRNAs in 1 μL samples, with a resolution of 3.55 × 10^−15^ M. This visualization analysis of ultratrace biomarkers based on acoustic enrichment and promotion provides a new perspective for rapid and highly sensitive clinical detection of Alzheimer’s disease. According to the research of Fleming Dackson Gudaguti et al. [99], negative dielectrophoresis (DEP) spectroscopy serves as an effective transduction mechanism for a biosensor designed to detect single nucleotide polymorphisms (SNPs) in short DNA strands. They observed a frequency-dependent negative DEP force applied by interdigitated electrodes to polystyrene microspheres (PMs) in response to changes in both the last and second-to-last nucleotides of a single-strand DNA bound to the PM. The drift velocity of the PM functionalized with single-strand DNA, which is directly proportional to the DEP force, was measured within a frequency range of 0.5 MHz to 2 MHz. The drift velocity was calculated using custom-made automated software employing real-time image processing techniques. This SNP genotyping technology holds promise for use in diagnosing and identifying genetic variants associated with diseases.

A summary of the applications of fluorescent microspheres in genome detection reveals that these microspheres have demonstrated considerable potential in genome nucleic acid detection. Firstly, they can simultaneously detect multiple genes or loci with high throughput and high parallelism, thereby enhancing detection efficiency and sample processing speed. Secondly, fluorescent microsphere technology boasts extremely high sensitivity and specificity, enabling the detection of target nucleic acids at low concentrations, which is beneficial for early disease diagnosis and monitoring. Furthermore, fluorescent microspheres can also achieve multiple reactions, which means detecting multiple targets simultaneously in the same sample, including gene mutations, single nucleotide polymorphisms, and so forth. This is of great significance for the development of personalized medical treatment and disease classification. Additionally, fluorescent microsphere technology has the advantages of low cost and easy operation, and is suitable for clinical diagnosis, gene typing, and other fields. In conclusion, the potential of fluorescent microspheres in genomic nucleic acid testing is considerable, with the possibility of facilitating the advancement of precision medicine and personalized treatment.

### 3.3. Application of Fluorescent Microspheres in High-Throughput Drug Screening

**Figure 5:**
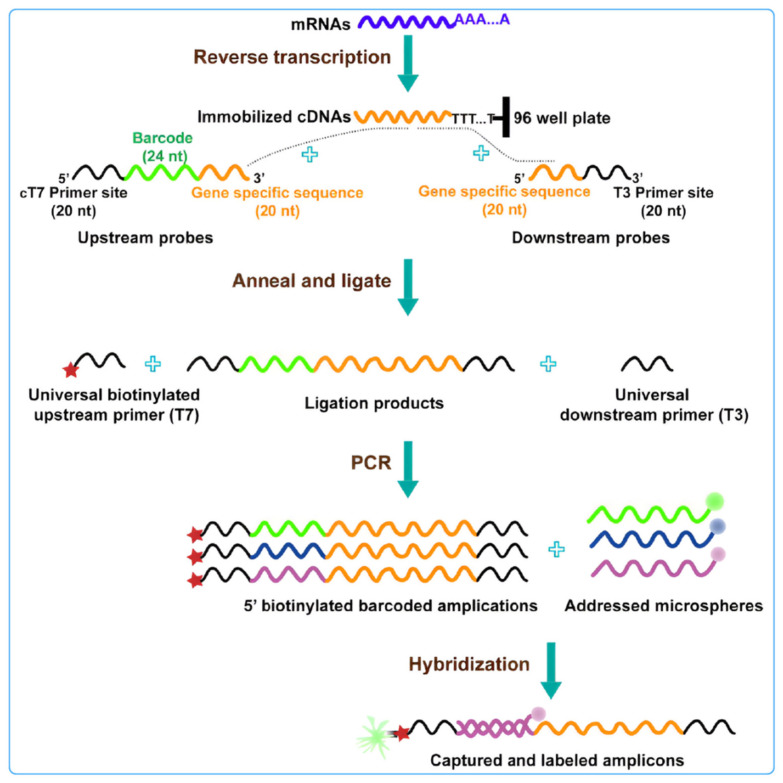
Schematic diagram of L1000 strategy technology: this technology uses connection mediated amplification and hybridization to Luminex beads, highlighting changes in gene expression by detecting bead color and fluorescence intensity of leaf green protein signals [100].

The application of fluorescent microspheres in the L1000 strategy involves the simultaneous detection of multiple gene expression levels by combining different fluorescent labels with specific gene probes [100]. Initially, RNA is extracted from test samples and transcribed into complementary DNA (cDNA). Subsequently, the cDNA is mixed with fluorescent microspheres containing thousands of gene probes, ensuring that each gene probe binds to a specific fluorescent microsphere. The mixed solution was then subdivided into multiple regions, each containing a distinct fluorescent microsphere, using a mixing approach. Using a Luminex instrument, the fluorescence intensity of each fluorescent microsphere in each region was measured, and the gene expression levels of each gene were determined. Finally, by comparing the fluorescence intensities across different samples, the impact of drugs or compounds on gene expression could be assessed [101] (Figure 5).

Tsung-Chieh Lin and colleagues [102] employed the L1000 strategy to identify drugs for the treatment of renal cell carcinoma (RCC). DDX3X, a protein involved in RNA metabolism [103,104,105], was found to be epigenetically downregulated in RCC. Transcriptome analysis revealed that lower levels of DDX3X promote the expression of genes in the SPINK1-metallothionein pathway, resulting in tumor growth, metastasis, and poorer prognosis in RCC patients [105]. Based on the characteristics of the DDX3X gene and the L1000 dataset, digoxin was identified as a compound that can reverse the gene signature associated with low DDX3X levels, thereby inhibiting cell proliferation and metastasis [106]. Using the L1000 platform and GEO database, Yu-Min Huang and colleagues analyzed and proposed ciclopirox, an approved antifungal medication, as a potential novel inhibitor of HMGA2. Molecular docking further indicated the direct interaction between ciclopirox and the AT-hook motif of HMGA2. Functional assays demonstrated that ciclopirox inhibits colorectal cancer cell growth by inducing cell cycle arrest and apoptosis. Kuo-Chang Wen and his team [107] used cancer genome atlas datasets, L1000 microarray data with gene set enrichment analysis (GSEA), protein blot analysis, and animal models to investigate the synergy of the AKT/mTOR pathway in response to neoadjuvant metformin combined with chemotherapy. Their findings suggested that ovarian cancer patients treated with metformin had significantly longer overall survival than did those who did not receive metformin treatment. This research implies that neoadjuvant metformin at clinically relevant doses is effective for treating ovarian cancer and provides insights for guiding clinical trials. Peter Natesan Pushparaj and colleagues [108] employed the L1000FWD and L1000 feature direction signature search engine (L1000 CDS2) network tools to discover small molecules that may reverse gene signatures associated with COVID-19 and Neuro-COVID-19. Several small molecules, including withaferin A, importazole, and nariclasine, which potentially reverse gene signatures related to COVID-19, have been identified. Moreover, withaferin A, echinomycin A, nariclasine, ciclopirox, and JQ1 have the potential to reverse gene signatures associated with Neuro-COVID-19. Tsang Pai Liu et al. [109] integrated the next-generation L1000-based CMap and an analytic Web tool, the L1000FWD, for systematic analyses of polypharmacology and drug repurposing. Two different types of anticancer drugs, histone deacetylase (HDAC) inhibitors and topoisomerase inhibitors, were used as proof-of-concept examples. We identified KM-00927 and BRD-K75081836 as novel HDAC inhibitors and mitomycin C as a topoisomerase IIB inhibitor.

The application of fluorescent microspheres in the L1000 strategy represents a significant advance in the field of drug discovery and gene expression analysis. By simultaneously detecting thousands of genes with varying expression levels, fluorescence microsphere technology enables the rapid and efficient acquisition of a substantial quantity of gene expression data in a single experiment. This high-throughput approach allows researchers to gain a comprehensive understanding of the impact of drugs on gene expression, thereby accelerating the processes of drug screening and optimization. Furthermore, fluorescent microsphere technology exhibits high sensitivity and specificity, enabling the accurate detection of subtle alterations in gene expression. This capability is invaluable in the discovery of novel drug targets and the elucidation of the mechanisms of action of drugs. Consequently, the integration of fluorescent microspheres in the L1000 strategy offers a rapid and precise analytical instrument for the advancement of drug development. It is anticipated that this will facilitate the acceleration of new drug launches and the advancement of drug discovery.

### 3.4. Application of Fluorescent Microspheres in Tumor Nucleic Acid Detection

**Figure 6:**
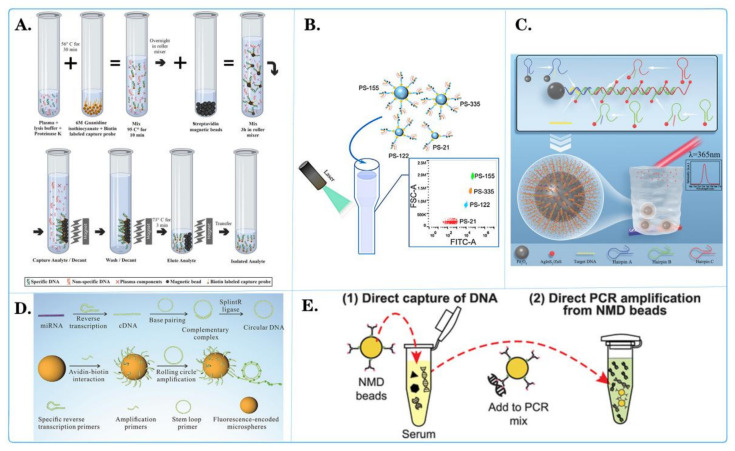
Fluorescent microspheres applied for tumor nucleic acid detection. (**A**) Schematic diagram of selective capture of plasma cell-less DNA on magnetic beads [118]. (**B**) Schematic diagram of the binding of central microspheres with multiple molecules and their interaction with pipettes containing other elements [111]. (**C**) Schematic diagram of high sensitivity fluorescence detection of CYFRA21-1 DNA in lung-cancer-based on quantum dot cumulative hybridization [120]. (**D**) Schematic diagram of quantitative detection of miRNAs related to triple-negative breast cancer by rolling circle sampling on fluorescent coding microspheres [110]. (**E**) Schematic diagram of using nitrogen-mustard-coated microbeads to directly capture and amplify small DNA fragments [117].

Fluorescent microspheres are nanoparticles with wide application potential and have also been widely studied in the field of tumor nucleic acid detection (Figure 6). They can be combined with other detection technologies, such as PCR, LAMP, and electrochemical detection, to construct a multifunctional detection platform. This combination can play a mutually complementary role in tumor nucleic acid detection and can improve the reliability of the results while being fast and sensitive.

Jieyu Liu and colleagues [110] developed a method to quantitatively detect the specific expression of multiple miRNAs, including miR-16, miR-21, miR-92, miR-199, and miR-342, in triple-negative breast cancer (TNBC) using rolling circle amplification (RCA) on fluorescent-encoded microspheres (Figure 6D). The experimental results demonstrated that the expression profiles of these specific miRNAs in TNBC can serve as early diagnostic markers. Early diagnosis of TNBC through these biomarkers can contribute to decreasing mortality rates. Ningning Wang and collaborators [111] engineered a novel polystyrene (PS) microsphere array to simultaneously identify and quantify various cancer-related microvesicles using a flow cytometer (Figure 6B). This microsphere array consisted of chain-modified PS beads, biotinylated substrate chains for 17-8Dnazyme (17S), and two split parts of the Dnazyme (PA, PB). The 17S was tagged with 6-carboxyfluorescein (FAM) and Dabcyl on either side of the ribonucleic acid. When target microvesicles are present, they can bind to their corresponding PAs and PBs, forming the active secondary structure of the Dnazyme. Active Dnazyme cleaves Dabcyl on the bead surface, restoring the fluorescence intensity. Additionally, the released target microvesicles could autonomously move to nearby inactive Dnazymes, amplifying the fluorescence signal. By reading the fluorescence output from the flow cytometer, the presence of target microvesicles could be quantified. The microsphere array successfully captured and quantified reaction materials related to cancer, such as MIR-21, MIR-155, MIR-335, and MIR-122, under buffer and serum conditions, demonstrating excellent selectivity and high sensitivity. Shutong Wang and colleagues [112] explored the potential of three circulating microRNAs (MR-21, MR-16, and MR-100) as diagnostic markers for liver cancer. They prepared dual-peak and dual-core magnetic microspheres for total RNA purification in serum. Concurrently, real-time quantitative PCR was used to detect microRNAs in the serum of liver cancer patients and healthy volunteers. The results showed differential MR-21 expression in the serum of liver cancer patients. In the population of Guangdong Province, China, MR-21, in conjunction with alpha-fetoprotein, could serve as a diagnostic marker for liver cancer. This method yields high-quality total RNA, demonstrating great potential for application. Currently, circulating cell-free DNA (cfDNA) in liquid biopsies is considered a crucial biomarker for cancer diagnosis. However, due to its small fragment size and low concentration in circulation, extracting and purifying cfDNA from serum samples has become complex, affecting the accuracy of subsequent molecular diagnostic tests [113,114,115,116]. Benediktus N Hapsianto and colleagues [117] introduced a new method using nitrogen-mustard-coated DNA capture beads (NMD beads) that allows direct PCR amplification of the beads without the need for elution (Figure 6E). To illustrate the diagnostic application of NMD beads, short DNA fragments implanted in bovine serum were tested. These implanted DNAs could be directly captured from serum samples and detected using real-time PCR, even at concentrations as low as 10 fg/mL. Therefore, this DNA capture bead technology is expected to simplify the preprocessing steps for cfDNA detection, expanding the diagnostic application of liquid biopsies. Mohammad Amin Kerachian and colleagues [118] developed and optimized a selective method for capturing cfDNA on magnetic beads for mutation detection in the plasma of colorectal cancer patients [119] (Figure 6A). The SCC-MAG method exhibited lower detection limits than did the silicon membrane method and enabled accurate determination of ctDNA content in plasma. Through experimentation, the SCC-MAG method could capture ctDNA from all samples, while the silicon membrane method could only collect ctDNA from some samples. Consequently, the SCC-MAG method proves to be a robust, replicable, and highly sensitive approach for analyzing mutation status in liquid biopsies, facilitating the diagnosis of multiple targets through the reuse of multiple probes. Ling Yang and colleagues [120] synthesized hydrophilic AgInS2@ZnS core-shell quantum dots and magnetic Fe_3_O_4_ nanoparticles (Figure 6C). They employed a hybridization chain reaction triggered by ctDNA to detect lung-cancer-related CYFRA21-1 DNA [121]. In the presence of CYFRA21-1 DNA, three hairpin structures were successively opened, leading to the accumulation of quantum dots and a significant change in the fluorescence signal. Compared to traditional fluorescence detection methods, Fe_3_O_4_ exhibits magnetic adsorption properties and a larger surface area and is suitable for immobilizing and aggregating quantum dot nanoparticles attached to single-stranded DNA. The concentration of CYFRA21-1 closely correlated with the number of remaining quantum dots after magnetic adsorption, providing a promising method for quantitative ctDNA detection.

Fluorescent microspheres are of great significance in the field of tumor nucleic acid detection. Their high specificity and sensitivity permit the accurate detection of tumor markers or mutated genes, thereby providing a reliable basis for early diagnosis, treatment selection, and monitoring treatment efficacy. Concurrently, the attributes of multiplex detection, straightforward operation, and the capacity to visualize outcomes render them irreplaceable instruments in tumor research and clinical applications. They furnish technical support for personalized medical care and contribute to the enhancement of survival rates and the quality of life of cancer patients. It is anticipated that, as technology continues to evolve and the scope of applications expands, fluorescent microspheres will facilitate the development of more efficient and accurate methods for the detection of tumor nucleic acids, thereby providing enhanced support for the implementation of personalized medicine and precision treatment.

## 4. Conclusions

Multiplex nucleic acid testing plays a crucial role in society today. It is widely used for diagnosing and tracking new coronaviruses during pandemics [122,123,124], and it plays a key role in various medical treatments and disease control, making it one of the indispensable technologies in society today, contributing greatly to the development of health and science [125,126,127]. However, the current problems mainly include challenges in accuracy, specificity, and sample handling. The use of fluorescent microspheres as novel nanomaterials solves several key problems for multiple nucleic acid detection. First and foremost is their superior sensitivity, which allows them to detect extremely low concentrations of nucleic acids, critical for accurate diagnosis of early-stage disease. In addition, fluorescent microsphere technology has the capability for multichannel detection, allowing simultaneous detection of multiple nucleic acid targets, effectively improving detection efficiency and shortening diagnostic time. Moreover, the risk of false alarms and missed alarms can be reduced through the rational selection of fluorescent labels and specific probe design, guaranteeing the accuracy of detection. Moreover, the combined application of fluorescent microsphere technology and automated systems simplifies sample processing steps [128], reduces the variability of human operation, and improves the consistency and reliability of the data.

Although fluorescent microsphere technology has enabled significant progress in multiple nucleic acid detection, there are still some challenges and problems to overcome. First, although the sensitivity of these methods has substantially improved, further improvements are needed to meet the detection needs of several diseases or low-concentration samples. To this end, consideration can be given to utilizing more sensitive fluorescent markers or fluorescent materials to increase the intensity of the signals, or further exploring emerging fluorescence detection technologies, such as nanoparticle- or fluorescent-phosphorescence-based methods, to further enhance the sensitivity of the assay. Second, specificity problems still exist in multiplexed nucleic acid detection, especially when multiple targets are detected at the same time. It is prone to cross-reactivity and better control methods are needed. Currently, technicians are working on the design of highly specific primers, multichannel detection and further optimization of reaction conditions to reduce nonspecific reactions and improve the accuracy of the assay. In addition, microsphere particles labeled with fluorescence may cause signal overlap and interference, for which researchers are also developing more advanced optical and analytical techniques to improve the clarity and interpretability of the data [129]. Finally, standardization and quality control issues are challenging, and ensuring the consistency of results between different laboratories and equipment is important. Addressing these challenges will require further research and technological innovation to continually improve the feasibility and reliability of fluorescent microsphere technology for multiplexed nucleic acid assays.

Nevertheless, it is anticipated that fluorescent microsphere technology will continue to offer significant potential for further development, a trend that will be enhanced by the ongoing advancements in nanotechnology and bioinformatics. It is likely that fluorescent microspheres will play a pivotal role in the field of multiple nucleic acid detection in the near future, with the following key trends emerging:

(1) With the continuous evolution of nanotechnology, fluorescent microsphere technology will become more intelligent. Intelligent microsphere systems will have the ability to automate sample processing, real-time data analysis, and instantaneous result reporting, thus greatly reducing human intervention and operational errors. This approach will make nucleic acid testing easier, faster, and more reliable.

(2) As the field of bioinformatics continues to advance, fluorescent microsphere technology will be better integrated with big data and bioinformatics. Tools such as high-throughput sequencing technology, data mining algorithms, and cloud computing will be widely used to enable more in-depth nucleic acid analysis and personalized medicine. Doctors and researchers will be able to provide customized medical solutions for each patient based on individual genetic information and disease characteristics.

(3) The application of fluorescent microsphere technology in personalized medicine and drug discovery will continue to expand. Individualized treatment will become a major trend in the future of medical care, and fluorescent microspheres will play a key role in helping doctors to better understand patients’ genomes and disease markers to provide more accurate diagnosis and treatment. In terms of drug development, fluorescent microspheres can be used to screen potential drug molecules and monitor their effects on biomarkers, providing a more effective tool for the development of new drugs.

In summary, fluorescent microsphere technology will be at the forefront of science and technology in the future, providing great opportunities in the fields of infectious disease outbreaks, public health safety and food safety incidents, customs inspection and quarantine, rare neonatal genetic diseases, and malignant tumor diagnosis. As nanotechnology and bioinformatics continue to evolve, this technology will become smarter, more efficient, and more versatile, providing us with more opportunities to improve health, treat disease, and advance medical research.

## Figures and Tables

**Table 1:** Comparison of preparation methods for fluorescent microspheres.

Method	Advantages	Disadvantages
Adsorption method	Simplified process. Ability to choose various fluorescent dyes. Can adjust fluorescence intensity.	Low stability. Low adsorption efficiency.
Embedding method	Enhances fluorescence stability. Performance can be controlled. Can form unique code.	Uneven fluorescence intensity. It will have an impact on fluorescent dyes. The cost in terms of time and money is high.
Self-assembly method	Simple preparation process. Morphology, size, and fluorescence properties of microspheres can be controlled. Offers good stability and long-term reliability.	Interaction force between particles and fluorescent materials must be considered. Operator needs advanced technical skills. The morphology and fluorescence properties of microspheres are unstable.
Chemical bonding method	Enhances signal stability and reliability. Enhances connection stability and durability. Improves uniformity of fluorescence signal and detection accuracy.	Sufficient dye binding sites are required. Dyes may fall off. The fluorescence properties are unstable.
Copolymerization method	The preparation process is straightforward, convenient, and economical. Stable fluorescence performance. Has different colors and fluorescence intensities.	The fluorescence intensity decreases. Dye detachment or decomposition leads to fluorescence instability. Limited application.
